# Attention needed for cognitive problems in patients after out-of-hospital cardiac arrest: an inventory about daily rehabilitation care

**DOI:** 10.1007/s12471-018-1151-z

**Published:** 2018-09-13

**Authors:** L. W. Boyce, P. H. Goossens, G. Volker, H. J. van Exel, T. P. M. Vliet Vlieland, L. van Bodegom-Vos

**Affiliations:** 1 Rijnlands Rehabilitation Centre, Leiden, The Netherlands; 2 Department of Biomedical Data Sciences, section Medical Decision Making, Leiden University Medical Center, Leiden, The Netherlands; 3 Department of Cardiology, Leiden University Medical Center, Leiden, The Netherlands; 4 Department of Orthopaedics, Rehabilitation and Physical Therapy, Leiden University Medical Center, Leiden, The Netherlands

**Keywords:** Out-of-hospital cardiac arrest, Rehabilitation care, Cognitive problems, Care pathway

## Abstract

**Aim:**

Recent literature and Dutch guidelines for patients with out-of-hospital cardiac arrest (OHCA) recommend screening for cognitive impairments and referral to cognitive rehabilitation when needed. The aim of this study is to assess the uptake of these recommendations for OHCA patients.

**Method:**

An internet-based questionnaire was sent to 74 cardiologists and 143 rehabilitation specialists involved in rehabilitation of OHCA patients in the Netherlands. The questionnaire covered: background characteristics, availability and content of cognitive screening and rehabilitation, organisation of care, experienced need for an integrated care pathway including physical and cognitive rehabilitation, barriers and facilitators for an integrated care pathway.

**Results:**

Forty-five questionnaires were returned (16 cardiologists and 29 rehabilitation doctors). Thirty-nine percent (*n* = 17) prescribed cognitive screening. Eighty-nine percent underscores an added value of an integrated care pathway. Barriers for an integrated care pathway included lack of knowledge, logistic obstacles, and poor cooperation between medical specialties.

**Conclusions:**

In the Netherlands, only a minority of cardiologists and rehabilitation specialists routinely prescribe some form of cognitive screening in OHCA patients, although the majority underscores the value of cognitive screening in OHCA patients in an integrated care pathway. The uptake of such a care pathway seems hindered by lack of knowledge and organisational barriers.

## What’s new

- MoCA should be administered routinely after out-of-hospital cardiac arrest (OHCA).
- A minority of cardiologists and rehabilitation specialists routinely screen for cognitive problems in OHCA patients.
- A majority of specialists underscores the value of cognitive screening in OHCA patients.
- Cooperation between cardiac and cognitive rehabilitation is needed.

## Introduction

The majority of patients who survive an out-of-hospital cardiac arrest (OHCA) are eligible for cardiac rehabilitation due to the cardiac cause of the arrest [1–5]. Cardiac rehabilitation focusses on physical activity, health education and stress management [6]. However, cardiac rehabilitation does not address the highly prevalent cognitive problems that 45–52% of the OHCA survivors experience [7].

Most common cognitive problems in OHCA patients, due to hypoxic brain injury, are memory problems, attention deficits and executive problems [7]. Cognitive problems, however mild, can have a major impact on a person’s participation/autonomy and quality of life and hamper good recovery [8]. Cognitive rehabilitation for patients with brain injury is proven effective [9].

In 1996, Grubb recommended paying attention towards cognitive problems after OHCA, subsequently emphasised by Moulaert in 2010 [10, 11]. Through the years guidelines also advised to screen for cognitive impairments and refer to cognitive rehabilitation when needed: the Dutch guidelines for cardiac rehabilitation of 2011 and the European Resuscitation Council (ERC) Guidelines for Resuscitation 2015 [5, 12–14]. One of the recommendations is to screen for cognitive impairments by using the Montreal Cognitive Assessment (MoCA) and to refer to a rehabilitation specialist if cognitive problems are found [12–14].

Additionally, the ERC mentions the use of the subjective Checklist for Cognition and Emotion as another possibility to identify possible cognitive symptoms. However, literature suggests that the use of subjective questionnaires merely discovers emotional problems [15]. Patients after OHCA frequently also suffer from emotional problems, such as anxiety (13–42%) and depression (8–45%) [16]. Emotional and cognitive symptoms often occur together and lead to poorer implementation of lifestyle changes and lower levels of participation [17]. A rehabilitation combination that covers both cardiac and neurological aspects seems the best option to reduce symptoms and improve patients’ well-being [16, 18, 19].

This article aims to assess the uptake of the recommendations regarding cognitive screening and rehabilitation in OHCA survivors as described in literature and recent guidelines and to assess barriers and facilitators that influence the uptake of these recommendations for OHCA survivors. Insight in the uptake of these recommendations is needed to enhance future initiatives that aim to improve the quality of care delivered to OHCA survivors.

## Materials and methods

### Setting

A majority of the 79 hospitals in the Netherlands provides cardiac rehabilitation to low-risk patients, supervised by a cardiologist. Cardiac rehabilitation for high-risk patients and/or cognitive rehabilitation are provided in 35 specialised rehabilitation centres with in total 150 locations under supervision of a rehabilitation specialist. Twenty-nine locations provide both cognitive and cardiac rehabilitation, 33 locations provide only cognitive rehabilitation and 6 provide only cardiac rehabilitation.

### Study design

In May 2015, an internet-based questionnaire was sent to rehabilitation specialists and cardiologists in Dutch rehabilitation centres and hospitals that provide cardiac and/or cognitive rehabilitation. Since most rehabilitation centres and hospitals were staffed with several medical specialists, multiple questionnaires were sent to each location. Reminders were sent two months later. The questionnaire consisted of open-ended, multiple choice or multi response questions. Questionnaires were returned anonymously.

### Study population

#### Cardiologists

Locations providing cardiac rehabilitation were retrieved by an internet search. Cardiologists registered with the Netherlands Society of Cardiology (*Nederlandse Vereniging voor Cardiologie—NVVC*) and the Dutch multidisciplinary consultative body on cardiac rehabilitation (*Landelijk Multidisciplinair Overleg Hartrevalidatie—LMDO-H*) were invited. Locations without a member of the Netherlands Society of Cardiology were invited to participate via a general email address. In total, 74 cardiologists were invited.

#### Rehabilitation specialists

All rehabilitation centres and locations were found via the website of *Revalidatie Nederland*, the Dutch branch organisation for rehabilitation centres. Cognitive rehabilitation specialists were traced via the *Werkgroep CVA Nederland *(WCN), a national workgroup on the rehabilitation of stroke patients. Locations not represented in the task force were invited to participate via a general email address. In total, 143 rehabilitation specialists were invited.

### Questionnaire

The questionnaire was based on review of literature and available guidelines [7, 19, 20]. Barriers were explored based upon the framework of Grol and Wensing, which categorises barriers and facilitators for the uptake of innovations into five levels: characteristics of the innovation, the individual professional, the individual patient, social context, organisational context and economic & political context [21]. During the development of the questionnaire, three semi-structured telephone interviews were conducted (a cardiologist and a rehabilitation specialist from a rehabilitation centre and a hospital cardiologist) to explore the barriers and facilitators for the uptake of the recommendations regarding cognitive screening, as input for the questions of the questionnaire.

The questionnaire included 30 questions about: (1) background characteristics (age, gender, institution [hospital, rehabilitation centre], work experience [years], number of OHCA patients per year [number of patients]); (2) availability and content of cognitive screening (patient routinely screened for cognitive problems [yes/no] and if not available, how are cognitive problems detected [intake, neuropsychological assessment, observation by team], what is the content of screening [objective, subjective], who is responsible for screening [cardiologist, rehabilitation specialist, psychologist, specialised nurse/physician assistant, paramedic], and what are the policies used when cognitive problems were suspected [intake psychologist, intake social worker, start cognitive rehabilitation, other]); and (3) experienced need for integrated care pathway in which cognitive screening is included [yes/no], existing barriers and facilitators for an integrated care pathway (awareness cognitive problems, logistic factors, factors regarding population, effects for patients [yes/no]).

### Analysis

We used descriptive statistics to analyse the data gathered by the questionnaire. The interviews were analysed qualitatively. However, the data from the interviews were only used to develop our questionnaire. We did not report the results of the interviews in our manuscript. The descriptive statistics were used for characteristics and current care for all respondents and for cardiologists or rehabilitation specialists separately. Chi-square tests, Mann-Whitney U tests and unpaired t‑tests (SPSS 22 v.02) were used to test differences between groups where appropriate. In case of expected cell count less than five, Fisher’s exact test was used. *P*-values ≤0.05 were considered statistically significant.

## Results

### Characteristics of respondents

The characteristics of respondents are shown in Table 1.

**Table 1:** Characteristics of respondents on a questionnaire on care in OHCA

	Total respondents	Cardiologists	Rehabilitation specialists
	*n* = 45	*n* = 16	*n* = 29
Age (median, range)	42 (31–61)	38 (31–59)	44 (31–61)
*Years of clinical experience with OHCA*			
1–5	11 (24%)	4 (25%)	7 (24%)
5–10	16 (36%)	8 (50%)	8 (28%)
10–15	8 (18%)	1 (6%)	7 (24%)
>15	10 (22%)	3 (19%)	7 (24%)
*Institution*			
Hospital	24 (53%)	9 (56%)	15 (52%)
Rehabilitation centre	19 (42%)	6 (38%)	13 (45%)
Hospital and rehabilitation centre	2 (5%)	1 (6%)	1 (3%)
*Estimated number of OHCA patients seen per year* ^a^			
0–10	18 (40%)	4 (25%)	14 (48%)
10–20	10 (19%)	1 (6%)	9 (31%)
20–30	4 (9%)	3 (19%)	1 (3%)
>30	10 (22%)	7 (44%)	3 (10%)
Unknown	3 (7%)	1 (6%)	2 (7%)

*CR* cardiac rehabilitation, *OHCA* out-of-hospital cardiac arrest, *PA* physician assistant, *GP* general practitioner

^a^*p*-value ≤0.05

A total of 45 respondents completed the questionnaire (21% response rate). The median age of respondents was 42 years (range 31–61), the majority (*n* = 16) had 5–10 years working experience with OHCA patients and saw 0–10 OHCA patients per year (*n* = 18). Fifty-three percent (*n* = 24) worked in a hospital, 42% (*n* = 19) in a rehabilitation centre and 5% (*n* = 2) in both.

### Availability and content of cognitive screening and rehabilitation

Table 2 shows the availability and content of cognitive screening and rehabilitation.

**Table 2:** Availability and content cognitive screening and rehabilitation for OHCA patients

	*N* (%)	Cardiologists	Rehabilitation specialists
*Screening (n* *=* *44)*		*n* = 16	*n* = 28
Yes	17 (39%)	6 (37%)	11 (39%)
No	27 (61%)	10 (63%)	17 (61%)
*Content screening (n* *=* *17)*^*a*^		*n* = 6	*n* = 11
Objective screening	11 (65%)	4 (67%)	7 (64%)
Subjective screening	6 (35%)	3 (50%)	3 (27%)
*Who assesses screening (n* *=* *17)*^*a*^		*n* = 6	*n* = 11
Cardiologist	0 (0%)	0 (0%)	0 (0%)
Rehabilitation specialist	5 (29%)	0 (0%)	5 (45%)
Psychologist	6 (35%)	1 (17%)	5 (45%)
Specialised nurse/physician assistant	5 (30%)	4 (67%)	1 (9%)
Paramedic	4 (24%)	1 (17%)	3 (27%)
Other	1 (6%)	1 (17%)	0 (0%)
*Assessment cognitive problems by lack cognitive screening* ^*a*^		*n* = 10	*n* = 17
Intake for cognitive rehabilitation	11 (41%)	1 (10%)	10 (59%)
Neuropsychological assessment	2 (8%)	0 (0%)	2 (12%)
Observation of cognitive problems by team	14 (54%)	9 (90%)	5 (29%)
Other	3 (12%)	0 (0%)	3 (18%)
*Action in case of possible cognitive problems (n* *=* *17)*^*a*^		*n* = 6	*n* = 11
Intake psychologist	4 (24%)	1 (17%)	3 (27%)
Intake social worker	3 (18%)	3 (50%)	0 (0%)
Start cognitive rehabilitation	12 (71%)	3 (50%)	9 (82%)
Other	1 (6%)	0 (0%)	1 (9%)
*Cooperation cardiac and cognitive rehabilitation (n* *=* *44)*		*n* = 16	*n* = 28
Care pathway/co-operative agreements	5 (11%)	3 (19%)	2 (7%)
Cardiac and cognitive rehabilitation in same team	4 (9%)	2 (13%)	2 (7%)
Easy referral from cardiac to cognitive rehabilitation	30 (68%)	11 (69%)	19 (68%)
NA/unknown	5 (11%)	0 (0%)	5 (18%)

*NA* not available, *OHCA* out-of-hospital cardiac arrest

^a^More than one answer possible

Of the 45 respondents 39% (*n* = 17) reported they used a standard cognitive screening in OHCA patients. Of these 17 respondents, 65% (*n* = 11) used an objective measurement: MoCA, Mini Mental State Examination (MMSE) or neuropsychological assessment.

Subjective methods are used by 35% (*n* = 6). A standardised questionnaire used by 2 of these respondents is the Cognitive Failures Questionnaire (CFQ). Cardiologists more often use a subjective screening (50%,* n* = 3) than rehabilitation specialists (27%, *n* = 3).

Cardiologists who reported using a screening, delegate this task to other health professionals. The majority of respondents who do not screen routinely rely on the observations during intake (41%, *n* = 11) or by their teams (54%,* n* = 14). In the absence of standard cognitive screening, cardiologists (90%, *n* = 9) use a non-structured observation, whereas rehabilitation specialists (59%, *n* = 10) refer patients to a cognitive rehabilitation team.

Respondents who screen for cognitive problems refer, when needed, to cognitive rehabilitation (71%, *n* = 12), psychologist (24%, *n* = 4) or social worker (18% *n* = 3).

Most respondents (68%, *n* = 30) find it is easy to refer from cardiac to cognitive rehabilitation in case of cognitive problems. Twenty percent has close collaborations in either a pathway (11%) or cardiac and cognitive rehabilitation within the same team (9%).

### Experienced need for an integrated care pathway and barriers and facilitators

Almost all respondents (89%, *n* = 39) see an added value in an integrated care pathway for OHCA patients (Tab. 3). One of the barriers mentioned for a care pathway is lack of knowledge of specialists regarding cognitive problems. However, most respondents are aware of memory problems (87%, *n* = 39), attention deficits (76%, *n* = 34), problems in reintegration in work (71%, *n* = 32) and relational problems (51%, *n* = 23). No major differences were found regarding awareness between responding cardiologists and rehabilitation specialists. Nevertheless, eight rehabilitation specialists (31%) mentioned a lack of knowledge regarding cognitive problems by cardiologists. Organisational barriers that hamper the implementation of a care pathway are logistic problems (44%, *n* = 17), difficulties in cooperation between cardiac and cognitive rehabilitation 36% (*n* = 14) and the small number of patients 36% (*n* = 14). In addition, 21% (*n* = 8) of the respondents fears an increase of administrative load and one person (3%) mentioned not achieving production agreements as a financial barrier.

**Table 3:** Barriers and facilitators for an integrated care pathway

		*n* (%)	Cardiologists	Rehabilitation specialists
***BARRIERS***				
**Knowledge** Lack of knowledge of cognitive problems by specialists for accurate referral		*n* = 42 8 (21%)	*n* = 16 0 (0%)	*n* = 26 8 (31%)
*Awareness cognitive impairment*		*n* = 45	*n* = 16	*n* = 29
Functioning	Memory problems	39 (87%)	14 (88%)	25 (86%)
	Attention deficits	34 (76%)	12 (75%)	22 (76%)
	Executive problems	16 (36%)	2 (13%)	14 (48%)
Activity	Decision making	12 (27%)	5 (31%)	7 (24%)
	Manage calendar	11 (24%)	3 (19%)	8 (28%)
	Make shopping list	4 (8%)	2 (13%)	2 (7%)
	Cleaning/running household	8 (18%)	3 (19%)	5 (17%)
Participation	Problems reintegration work	32 (71%)	13 (81%)	19 (66%)
	Relational problems	23 (51%)	10 (63%)	13 (45%)
	Safety	11 (24%)	1 (6%)	10 (34%)
	Attending traffic	10 (22%)	2 (13%)	8 (28%)
	Other	6 (13%)	2 (13%)	4 (14%)
**Organisational**		*n* = 42	*n* = 16	*n* = 26
Logistic problems		17 (44%)	5 (31%)	12 (46%)
Increase administrative load		8 (21%)	5 (31%)	3 (12%)
Patient population too small		14 (36%)	6 (38%)	8 (31%)
Difficulty cooperation departments		14 (36%)	3 (19%)	11 (42%)
**Financial**		*n* *=* *42*	*n* *=* *16*	*n* *=* *26*
Not achieving production agreements		1 (3%)	1 (6%)	0 (0%)
***FACILITATORS***				
**Organisational**		*n* = 44	*n* = 16	*n* = 28
*Existing cooperation between departments*				
Care pathway /co-operative agreements		5 (7%)	3 (19%)	2 (7%)
Cardiac and cognitive rehabilitation in same team		4 (9%)	2 (13%)	2 (7%)
Easy referral cardiac to cognitive rehabilitation		30 (50%)	11 (69%)	19 (68%)
NA/unknown		5 (11%)	0 (0%)	5 (18%)
*Added value care pathway/co-operative agreement*				
Yes		39 (89%)	15 (94%)	24 (86%)
No		5 (11%)	1 (6%)	4 (14%)
**Innovation**		*n* = 44	*n* = 15	*n* = 29
Care cardiac/neurorehabilitation better aligned		32 (78%)	10 (67%)	22 (76%)
Less drop outs cardiac rehab		1 (2%)	1 (7%)	0 (0%)
Less chance relapse		23 (56%)	9 (60%)	14 (48%)
Better alignment help request patient		25 (61%)	7 (47%)	18 (62%)

An opportunity is seen in the organisational facilitator of already existing co-operations between departments (89%). The majority (89%) sees an added value in a care pathway or co-operative agreement. Most respondents also see opportunities in better alignment of cardiac and cognitive rehabilitation (71%,* n* = 32), more focus on patients’ needs (61%, *n* = 25), fewer chances of relapse (56%, *n* = 23) and less dropouts during the cardiac rehabilitation programme (2%,* n* = 1).

## Discussion

This study describes that both cardiologists and rehabilitation physicians in the Netherlands pay attention to cognitive problems in OHCA patients. The uptake of the recommendations to assess cognitive problems in OHCA survivors is poor though and needs improvement. Although all respondents in this study mention they use some sort of screening, only 39% of the respondents routinely use a standardised screening for cognitive problems in OHCA patients and a mere 25% use a standardised objective screening.

Two objective screening tools are used by a small amount of respondents—MoCA and MMSE. The MoCA, which only takes 10 min, measures memory, visuospatial abilities, executive functions, attention, concentration and orientation of the cognitive impairment spectrum, and with a sensitivity of 90% and specificity of 87% it is the best short screening available at the moment [22, 23].

The ERC resuscitation guidelines also recommend MoCA and advise referral to a neuropsychologist or rehabilitation specialist if signs and symptoms of cognitive impairments are found.

Subjective instruments (based upon patients’ own point of view) are also recommended in the ERC resuscitation guidelines. The Cognitive Failures Questionnaire (CFQ) is such an instrument and is used by some of the respondents who routinely screen for cognitive problems. The CFQ, a questionnaire about cognitive mistakes, is not specifically recommended in the ERC guidelines but is similar to the recommended Checklist Cognition and Emotion. Literature suggests that the use of the CFQ might not reveal cognitive deficits but merely emotional problems, and should therefore rather be used complementary to the objective screening for cognitive problems [15].

Respondents who do not standardly screen (61%) indicate that they assess cognitive problems using non-structured observations by the team. Patients they suspect of cognitive problems are referred for cognitive rehabilitation or neuropsychological assessment. It is well known that non-structured observations lead to false-negative results that can be avoided by using either structured observations or screenings [22].

The lack of treatment protocols for both screening and treatment of cognitive deficits after OHCA is striking, given the recommendations in the guidelines.

Cognitive rehabilitation has proven to be effective for patients with acquired brain injury [9]. Cognitive rehabilitation teaches people how to compensate for cognitive impairments and how to use resources to retain optimal participation in society. Psycho-education is offered to help the patient and the family learns how to cope with cognitive and emotional consequences of brain injury [24]. Although no studies are available on the effectiveness of cognitive rehabilitation for patients with hypoxic brain injury due to cardiac arrest, it is likely that OHCA survivors with cognitive impairments benefit from cognitive rehabilitation interventions in the same way patients with other types of acquired brain injury do [9, 20].

A positive aspect towards future treatment protocols is that all specialists are aware of one or more possible cognitive problems. The majority of the respondents sees an added value in an integrated care pathway resulting in better alignment of care, better fulfilment of the patient’s needs and decrease of the chance of relapse. Since the vast majority (89%) already has an existing co-operation between departments, rapid implementation of the recommendations should be possible.

However, logistic barriers and lack of structural cooperation between cardiac and cognitive rehabilitation hamper the uptake of these recommendations. Specialists also fear an increase of administrative load for a small population.

### Strengths and limitations

We extensively approached specialists involved in care for OHCA patients. By doing so, multiple specialists at one location were approached. Often only one of them reacted, explaining the low response rate. Probably, this introduces a selection bias with overrepresentation of specialists interested in cognitive problems.

## Conclusion

Rehabilitation care for OHCA patients is suboptimal in the Netherlands. Patients are not routinely screened for cognitive impairments. To reassure that attention is paid to cognitive problems the MoCA should be administered routinely and cooperation between cardiac and cognitive rehabilitation is needed.
